# Crystal structures of anhydrous and hydrated *N*-benzyl­cinchonidinium bromide

**DOI:** 10.1107/S2056989022005096

**Published:** 2022-05-17

**Authors:** Daron E. Janzen, Maya S. Butler, Eric W. Reinheimer

**Affiliations:** a Dept. of Chemistry & Biochemistry, St. Catherine University, 2004 Randolph Avenue, St. Paul, MN 55105, USA; b Rigaku Americas Corporation, 9009 New Trails Drive, The Woodlands, TX 77381, USA; University of Kentucky, USA

**Keywords:** quaternary ammonium salt of cinchonidine, hydrogen bonding, crystal structure

## Abstract

*N*-benzyl­cinchonidinium bromide, C_26_H_29_N_2_O^+^·Br^−^, with the systematic name (*R*)-[(2*S*,4*S*,5*R*)-1-benzyl-5-ethenyl-1-azoniabi­cyclo­[2.2.2]octan-2-yl](quinolin-4-yl)­methanol bromide, is a quaternary ammonium salt of the *cinchona* alkaloid cinchonidine. This salt is widely used as a chiral phase-transfer catalyst and chiral resolution agent. Both classical and non-classical hydrogen-bonding inter­actions, as well as anion effects have been shown to play key mechanistic roles in the catalysis of *cinchona* alkaloids. In an effort to understand the effects of water on these inter­molecular inter­actions, the structures of anhydrous *N*-benzyl­cinchonidinium bromide, (I), and the sesquihydrate, C_26_H_29_N_2_O^+^·Br^−^·1.5H_2_O, (II), were determined.

## Abstract 2

The crystal structures of anhydrous *N*-benzyl­cinchonidinium bromide and the sesquihydrate are reported. O—H hydrogen-bond donor inter­actions and numerous C—H⋯Br contacts dominate the inter­molecular features.

## 1. Chemical context

*Cinchona-*derived enanti­oselective phase-transfer catalysts have been used in a variety of applications including [2,3]-Wittig rearrangements (Denmark & Cullen, 2015 ▸), synthesis of unnatural α-amino acids (O’Donnell *et al.*, 1989 ▸), and even industrial-scale synthesis of pharmaceuticals (Moccia *et al.*, 2015 ▸). As this class of phase-transfer catalysts are easy to prepare from the parent natural product alkaloids, and demonstrate aspects of green and sustainable chemistry, they are attractive organocatalysts for further development. Mechanistic studies of *N*-benzyl­cinchonidinium bromide and substrates in solution provide evidence for the importance of quaternary ammonium benzylic C—H hydrogen-bond donor inter­actions as well as the classical OH donor (Bencivenni *et al.*, 2021 ▸). Anion effects also demonstrate differences in the binding mode of substrates with mechanistic implications and potential enanti­oselectivity. 
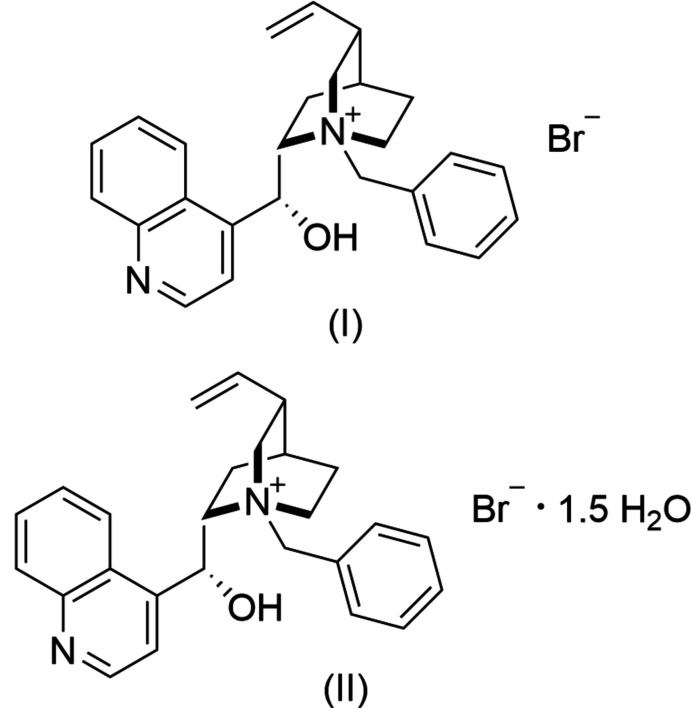


While structures are reported for analogs of this cation, that of the commercially available bromide salt is unpublished. We report here the structures of *N*-benzyl­cinchonidinium bromide (I) and the sesquihydrate (II).

## 2. Structural commentary

**Figure 1:**
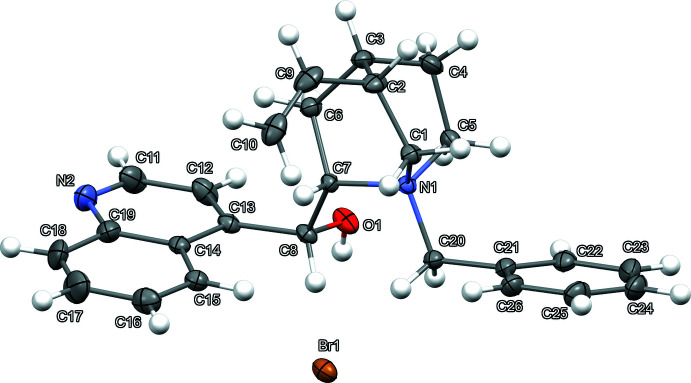
Mol­ecular structure of (I) with displacement ellipsoids drawn at the 50% probability level.

**Figure 2:**
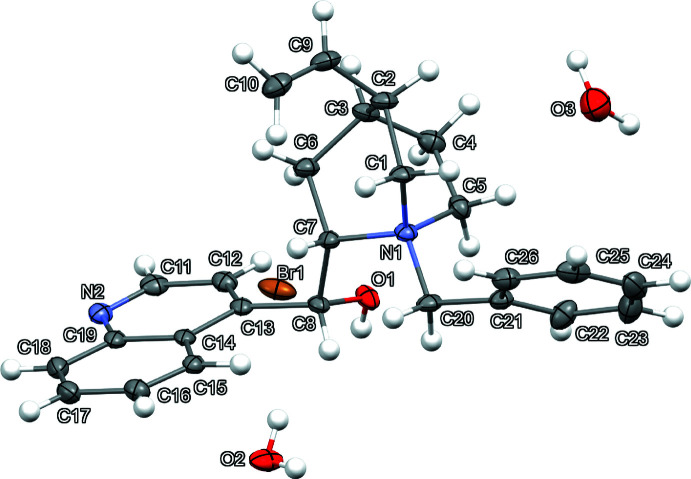
Mol­ecular structure of (II) with displacement ellipsoids drawn at the 50% probability level.

The anhydrous compound (I) (Fig. 1 ▸) crystallizes in the monoclinic space group *P*2_1_. The asymmetric unit of (I) consists of one mol­ecular cation and one bromide anion. The sesquihydrate (II) (Fig. 2 ▸) crystallizes in the tetra­gonal space group *P*4_1_2_1_2. The asymmetric unit of (II) consists of one mol­ecular cation, one bromide anion, and one water on a general position and one half water, as O3 lies on a twofold axis at *z* = 0.5. For (I) and (II), the absolute configuration of chiral atoms N1, C2, C3, C7, and C8 are determined as *S*, *R*, *S*, *S*, and *R*, respectively, by anomalous dispersion and are consistent with previous structures of cinchonidine.

**Figure 3:**
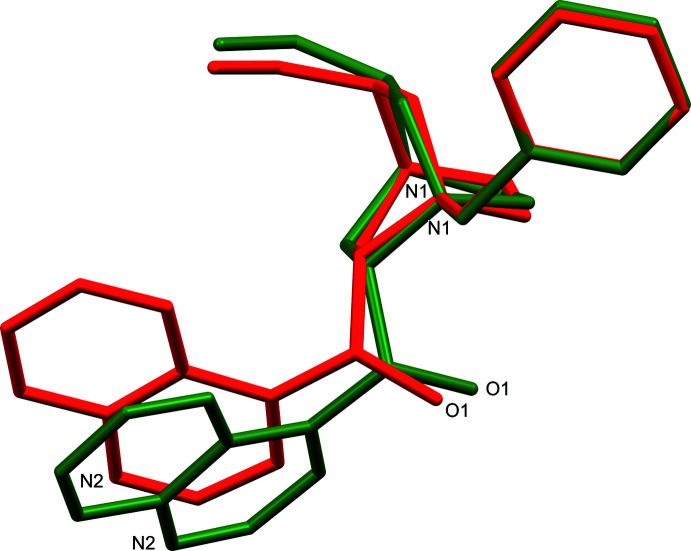
Overlap of quinuclidine non-H atom coordinates (C1–C7, N1) of the *N*-benzyl­cinchonidinium cation of (I) (red) and (II) (green).

**Figure 4:**
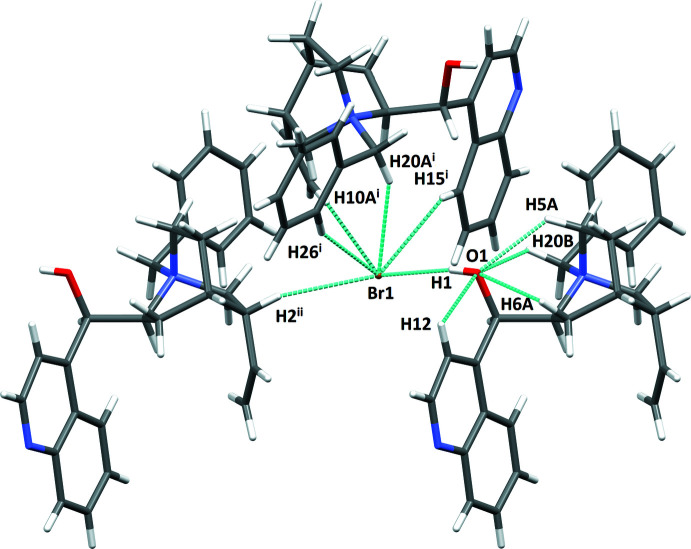
Intra- and inter­molecular inter­actions of (I). Symmetry codes: (i) 1 − *x*, *y* − 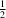 , 1 - *z;* (ii) 1 + *x*, *y*, *z*.

Most analogous bond lengths in (I) and (II) show only minor differences, with two exceptions (Tables 1 ▸ and 2 ▸). The largest differences in bond lengths occur for C6—C7 [1.510 (4) Å (I), 1.553 (8) Å (II)] and N2—C11 [1.282 (6) Å (I), 1.319 (9) Å (II)]. The quinuclidine intra­molecular N1⋯C3 distances show small expansion of this bicyclic ring system from (I) [2.534 (5) Å] to (II) [2.591 (8) Å]. Overlap of the *N*-benzyl­cinchonidinium cation atom coordinates of (I) and (II) (Fig. 3 ▸) shows significant conformational differences. While the quinuclidine, benzyl, and vinyl functionalities adopt very similar conformations for (I) and (II), larger changes are observed in the alcohol and quinoline groups. Torsion angles that highlight the largest conformational changes include C7—C8—C13—C12 [107.9 (3)° (I); 101.3 (7)° (II)], C8—C7—N1—C20 [−39.0 (3)° (I); −53.6 (7)° (II)], and O1—C8—C13—C12 [−11.7 (4)° (I); −19.2 (8)° (II)]. These torsion-angle differences result in large changes in the relative angles between least-squares planes of the phenyl and quinoline groups in (I) [14.8 (2)°] and (II) [41.8 (3)°]. Intra­molecular C—H⋯O contacts C5—H5*A*⋯O1 are found in both (I) and (II), but (I) shows an additional benzylic C20—H20*B*⋯O1 contact (Tables 3 ▸ and 4 ▸, Figs. 4 ▸ and 5 ▸).

## 3. Supra­molecular features

The extended structure of (I) displays a simple isolated charge-assisted hydrogen bond with the alcohol donor O1 and Br1 anion acceptor (Table 3 ▸, Fig. 4 ▸). The quinoline N2 acceptor does not participate in any hydrogen-bonding inter­actions. Each bromide also has four short C—H⋯Br contacts with the same cation (phenyl, benzyl, quinoline, and vin­yl) as well as an additional quinuclidine methine C—H.

**Figure 5:**
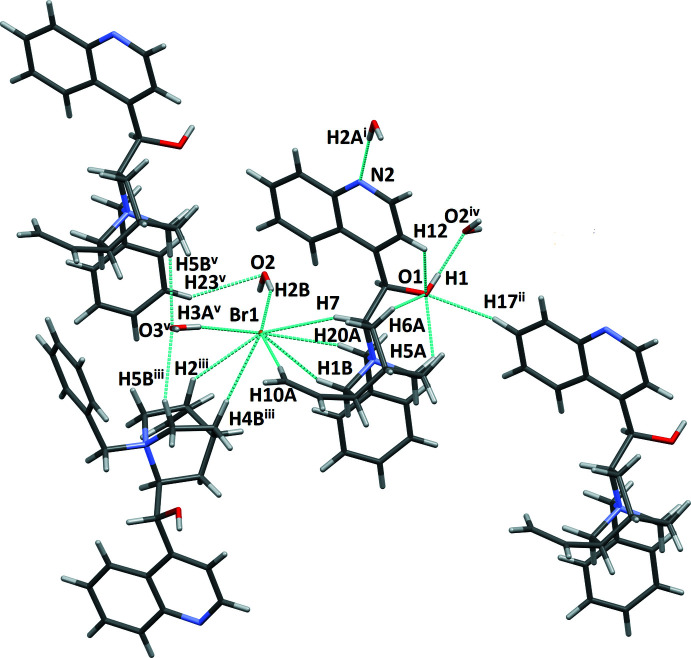
Intra- and inter­molecular inter­actions of (II). Symmetry codes: (i) *x*, 1 + *y*, *z*; (ii) 1 + *x*, *y*, *z*; (iii) *y*, −1 + *x*, 1 − *z*; (iv) 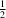 + *x*, 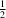 − *y*, 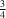 − *z*; (v) −1 + *x*, *y*, *z*

The sesquihydrate (II) shows very different hydrogen-bonding inter­actions (Table 4 ▸, Fig. 5 ▸). The alcohol group O1 acts as a donor with a water acceptor, O2. Water O2 hydrogen bonds as donor with Br1 and quinoline N2, while water O3 acts a donor to two bromide acceptors. This pattern of hydrogen bonds forms a chain with terminal O1 donors and water and bromide links, with the water O2 relating the two halves of the chain. Quinoline N2 acceptors of O2 hydrogen-bond donors link the chains forming an extended network. Each bromide also has four short C—H⋯Br contacts with the same cation (benzyl, vinyl, and two quinuclidine) as well as two additional quinuclidine contacts with a neighboring mol­ecular cation (Figs. 5 ▸ and 6 ▸).

## 4. Database survey

A search of the Cambridge Structural Database (*ConQuest* version 2022.1.0; Groom *et al.*, 2016 ▸) yields several related analogs of both *N*-benzyl­cinchonidinium salts as well as the pseudo-enanti­omer *N*-benzyl­cinchoninium. The 2-fluoro­benzyl bromide sesquihydrate analog XUNQIG (Jew *et al.*, 2002 ▸) is isostructural with (II) though additional C—H⋯F intra- and inter­molecular inter­actions are present. Introduction of the aromatic 2-fluoro substituent yielded enhanced enanti­oselectivity in catalytic phase-transfer alkyl­ation reactions, with possible origins related to more conformational or dipole changes to enhance substrate binding. Other closely related *N*-benzyl­cinchonidinium chloride salts have been employed in co-crystal resolution of a chiral spiro­cyclic diol (GAJBOJ01; Zhang *et al.*, 2006 ▸), atropisomeric chiral diols (HADSIS; Walsh *et al.*, 2021 ▸ and JAPGIR; Sweetman *et al.*, 2005 ▸) and a related mixed chiral amine/alcohol (GOSWIU; Ding *et al.*, 1999 ▸). Even in the presence of multiple additional hydrogen-bond donors in these co-crystals, short benzylic C—H⋯Cl contacts are retained in GAJBOJ01 and JAPGIR, though not in HADSIS or GOSWIU. The *N*-benzyl­cinchonidinium cation has also been employed in resolution of chiral halogenated phosphates (GARJUF, GAWSUT; Frantz *et al.*, 2005 ▸). Short benzylic C—H⋯O contacts are found in these chiral phosphate salts.

Closely related cinchoninium anhydrous bromide structures with phenyl substituents [2-bromo­benzyl, QEDZAC (Skórska-Stania *et al.* 2012 ▸) and 3,5-bis­tri­fluoro­methyl, UHINUV (Kawai *et al.*, 2009 ▸)] show similar O—H⋯Br hydrogen bonding to (I). However, the C—H⋯Br inter­actions differ. In QEDZAC, each bromide has quinuclidine, quinoline, and benzyl C—H⋯Br contacts with the same cation. In UHINUV, quinoline, benzyl, and phenyl C—H⋯Br contacts with the same cation are found. The *N*-benzyl­cinchoninium chloride salt has also been employed in a co-crystal resolution of BINOL (WOMQUK01; Walsh *et al.*, 2021 ▸).

## 5. Synthesis and crystallization

*N*-benzyl­cinchonidinium bromide was purchased from Sigma-Aldrich (St. Louis, Missouri, USA). Crystals of the anhydrous form (I) were obtained by vapor diffusion of diethyl ether into an aceto­nitrile solution of *N*-benzyl­cinchonidinium bromide. Crystals of the sesquihydrate (II) were obtained by slow evaporation of an ethanol solution of *N*-benzyl­cinchonidinium bromide.

## 6. Refinement

Crystal data, data collection and structure refinement details are summarized in Table 5 ▸. The O—H hydrogen positions were assigned from residual electron-density peaks and positions were refined. All remaining hydrogen atoms were placed in calculated positions and refined in the riding-model approximation with distances of C—H = 0.93, 0.93, 0.93, 0.97, and 0.98 Å for the aromatic C—H, terminal vinyl CH_2_, vinyl C9—H9, methyl­ene C—H, and methine C—H, respectively, and with *U*_iso_(H) = *k*·*U*_eq_(C), *k* = 1.2 for all C—H and 1.5 for the hydroxyl H1.

## Supplementary Material

Crystal structure: contains datablock(s) I, II. DOI: 10.1107/S2056989022005096/pk2664sup1.cif

Structure factors: contains datablock(s) I. DOI: 10.1107/S2056989022005096/pk2664Isup2.hkl

Structure factors: contains datablock(s) II. DOI: 10.1107/S2056989022005096/pk2664IIsup3.hkl

CCDC references: 2172120, 2172119

Additional supporting information:  crystallographic information; 3D view; checkCIF report

## Figures and Tables

**Figure 6:**
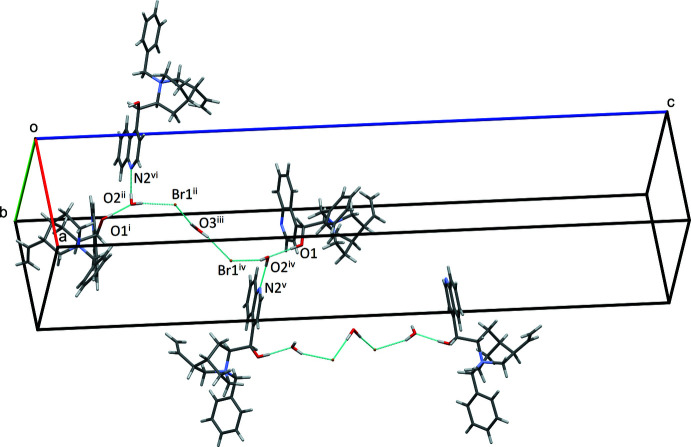
Inter­molecular hydrogen-bonding pattern of (II). Symmetry codes: (i) 1 − *y*, 1 − *x*, 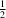 − *z*; (ii) 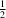 + *y*, 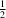 − *x*, − 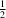 + *z*; (iii) 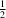 + *y*, 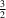 − *x*, − 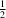 + *z*; (iv) 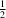 + *x*, 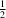 − *y*, 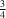 − *z*; (v) 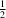 + *x*, 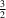 − *y*, 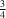 − *z*; (vi) − 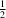 + *y*, 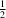 − *x*, − 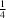 + *z*

**Table 1:** Selected geometric parameters (Å, °) for (I)

N2—C11	1.282 (6)	C6—C7	1.510 (4)
			
C12—C13—C8—O1	−11.7 (4)	C20—N1—C7—C8	−39.0 (3)
C12—C13—C8—C7	107.9 (3)		

**Table 2:** Selected geometric parameters (Å, °) for (II)

N2—C11	1.319 (9)	C7—C6	1.553 (8)
			
O1—C8—C13—C12	−19.2 (8)	C20—N1—C7—C8	−53.6 (7)
C7—C8—C13—C12	101.3 (7)		

**Table 3:** Hydrogen-bond geometry (Å, °) for (I)

*D*—H⋯*A*	*D*—H	H⋯*A*	*D*⋯*A*	*D*—H⋯*A*
O1—H1⋯Br1	0.73 (5)	2.45 (5)	3.149 (3)	162 (5)
C15—H15⋯Br1^i^	0.93	2.90	3.644 (4)	137
C12—H12⋯O1	0.93	2.39	2.739 (5)	102
C6—H6*A*⋯O1	0.97	2.58	2.967 (4)	104
C2—H2⋯Br1^ii^	0.98	2.83	3.779 (3)	164
C26—H26⋯Br1^i^	0.93	2.87	3.738 (4)	155
C5—H5*A*⋯O1	0.97	2.36	3.024 (4)	125
C20—H20*A*⋯Br1^i^	0.97	2.91	3.800 (3)	153
C20—H20*B*⋯O1	0.97	2.64	3.198 (4)	117
C10—H10*A*⋯Br1^i^	0.93	3.02	3.943 (4)	172

Symmetry codes: (i) 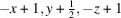 ; (ii) 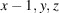 .

**Table 4:** Hydrogen-bond geometry (Å, °) for (II)

*D*—H⋯*A*	*D*—H	H⋯*A*	*D*⋯*A*	*D*—H⋯*A*
O1—H1⋯O2^i^	0.89 (8)	1.75 (8)	2.629 (7)	168 (8)
O2—H2*A*⋯N2^ii^	0.88 (10)	1.97 (10)	2.824 (8)	161 (9)
O2—H2*B*⋯Br1	0.75 (9)	2.48 (9)	3.202 (5)	160 (10)
C7—H7⋯Br1	1.00	2.99	3.894 (6)	151
C12—H12⋯O1	0.95	2.44	2.771 (8)	101
C2—H2⋯Br1^iii^	1.00	2.98	3.811 (7)	142
C1—H1*B*⋯Br1	0.99	2.88	3.779 (7)	152
C5—H5*A*⋯O1	0.99	2.29	2.836 (8)	114
C5—H5*B*⋯O3	0.99	2.56	3.464 (6)	151
C17—H17⋯O1^iv^	0.95	2.61	3.500 (8)	157
C6—H6*A*⋯O1	0.99	2.70	3.016 (8)	99
C4—H4*A*⋯Br1^iii^	0.99	2.94	3.785 (7)	144
C20—H20*A*⋯Br1	0.99	2.89	3.794 (7)	152
C10—H10*A*⋯Br1	0.95	3.01	3.960 (8)	176
C23—H23⋯O2^v^	0.95	2.71	3.518 (11)	143
O3—H3*A*⋯Br1^iii^	0.90 (10)	2.61 (10)	3.499 (6)	170 (11)

Symmetry codes: (i) 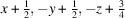 ; (ii) 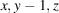 ; (iii) 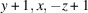 ; (iv) 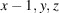 ; (v) 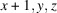 .

**Table 5:** Experimental details

	(I)	(II)
Crystal data		
Chemical formula	C_26_H_29_N_2_O^+^·Br^−^	2C_26_H_29_N_2_O^+^·2Br^−^·3H_2_O
*M*_r_	465.42	984.89
Crystal system, space group	Monoclinic, *P*2_1_	Tetragonal, *P*4_1_2_1_2
Temperature (K)	173	173
*a*, *b*, *c* (Å)	11.2574 (7), 8.8445 (5), 11.9039 (9)	9.9254 (2), 9.9254 (2), 47.1267 (14)
α, β, γ (°)	90, 110.126 (8), 90	90, 90, 90
*V* (Å^3^)	1112.85 (14)	4642.6 (2)
*Z*	2	4
Radiation type	Mo *K*α	Mo *K*α
μ (mm^−1^)	1.87	1.80
Crystal size (mm)	0.61 × 0.25 × 0.15	0.52 × 0.36 × 0.36

Data collection		
Diffractometer	XtaLABmini	XtaLABmini
Absorption correction	Multi-scan (*CrysAlis PRO*; Rigaku OD, 2020 ▸)	Multi-scan (*CrysAlis PRO*; Rigaku OD, 2020 ▸)
*T*_min_, *T*_max_	0.610, 1.000	0.281, 1.000
No. of measured, independent and observed [*I* > 2σ(*I*)] reflections	15118, 7531, 5890	36339, 4154, 3919
*R*_int_	0.030	0.078
(sin θ/λ)_max_ (Å^−1^)	0.765	0.597

Refinement		
*R*[*F* ^2^ > 2σ(*F* ^2^)], *wR*(*F* ^2^), *S*	0.041, 0.084, 1.01	0.051, 0.111, 1.05
No. of reflections	7531	4154
No. of parameters	274	297
No. of restraints	1	0
H-atom treatment	H atoms treated by a mixture of independent and constrained refinement	H atoms treated by a mixture of independent and constrained refinement
Δρ_max_, Δρ_min_ (e Å^−3^)	0.54, −0.29	0.35, −0.40
Absolute structure	Flack *x* determined using 2185 quotients [(*I* ^+^)−(*I* ^−^)]/[(*I* ^+^)+(*I* ^−^)] (Parsons et al., 2013 ▸)	Flack *x* determined using 1347 quotients [(*I* ^+^)−(*I* ^−^)]/[(*I* ^+^)+(*I* ^−^)] (Parsons et al., 2013 ▸)
Absolute structure parameter	−0.011 (5)	0.005 (7)

Computer programs: *CrysAlis PRO* (Rigaku OD, 2020 ▸), *SHELXT* (Sheldrick, 2015*a* ▸), *SHELXL* (Sheldrick, 2015*b* ▸), and *OLEX2* (Dolomanov *et al.*, 2009 ▸).

## supplementary crystallographic information

### (R)-[(2S,4S,5R)-1-Benzyl-5-ethenyl-1-azoniabicyclo[2.2.2]octan-2-yl](quinolin-4-yl)methanol bromide (I) Crystal data

**Table:**

C_26_H_29_N_2_O^+^·Br^−^	*F*(000) = 484
*M**_r_* = 465.42	*D*_x_ = 1.389 Mg m^−^^3^
Monoclinic, *P*2_1_	Mo *K*α radiation, λ = 0.71073 Å
*a* = 11.2574 (7) Å	Cell parameters from 8428 reflections
*b* = 8.8445 (5) Å	θ = 2.2–32.9°
*c* = 11.9039 (9) Å	µ = 1.87 mm^−^^1^
β = 110.126 (8)°	*T* = 173 K
*V* = 1112.85 (14) Å^3^	Block, colourless
*Z* = 2	0.61 × 0.25 × 0.15 mm

### (R)-[(2S,4S,5R)-1-Benzyl-5-ethenyl-1-azoniabicyclo[2.2.2]octan-2-yl](quinolin-4-yl)methanol bromide (I) Data collection

**Table:**

XtaLABmini diffractometer	7531 independent reflections
Radiation source: fine-focus sealed X-ray tube, Enhance (Mo) X-ray Source	5890 reflections with *I* > 2σ(*I*)
Graphite monochromator	*R*_int_ = 0.030
ω scans	θ_max_ = 32.9°, θ_min_ = 1.8°
Absorption correction: multi-scan (CrysAlisPro; Rigaku OD, 2020)	*h* = −16→16
*T*_min_ = 0.610, *T*_max_ = 1.000	*k* = −12→13
15118 measured reflections	*l* = −18→17

### (R)-[(2S,4S,5R)-1-Benzyl-5-ethenyl-1-azoniabicyclo[2.2.2]octan-2-yl](quinolin-4-yl)methanol bromide (I) Refinement

**Table:**

Refinement on *F*^2^	Hydrogen site location: mixed
Least-squares matrix: full	H atoms treated by a mixture of independent and constrained refinement
*R*[*F*^2^ > 2σ(*F*^2^)] = 0.041	*w* = 1/[σ^2^(*F*_o_^2^) + (0.0383*P*)^2^ + 0.0838*P*] where *P* = (*F*_o_^2^ + 2*F*_c_^2^)/3
*wR*(*F*^2^) = 0.084	(Δ/σ)_max_ = 0.001
*S* = 1.01	Δρ_max_ = 0.54 e Å^−^^3^
7531 reflections	Δρ_min_ = −0.29 e Å^−^^3^
274 parameters	Absolute structure: Flack *x* determined using 2185 quotients [(*I*^+^)-(*I*^-^)]/[(*I*^+^)+(*I*^-^)] (Parsons et al., 2013)
1 restraint	Absolute structure parameter: −0.011 (5)
Primary atom site location: dual	

### (R)-[(2S,4S,5R)-1-Benzyl-5-ethenyl-1-azoniabicyclo[2.2.2]octan-2-yl](quinolin-4-yl)methanol bromide (I) Special details

**Table:**

Geometry. All esds (except the esd in the dihedral angle between two l.s. planes) are estimated using the full covariance matrix. The cell esds are taken into account individually in the estimation of esds in distances, angles and torsion angles; correlations between esds in cell parameters are only used when they are defined by crystal symmetry. An approximate (isotropic) treatment of cell esds is used for estimating esds involving l.s. planes.

### (R)-[(2S,4S,5R)-1-Benzyl-5-ethenyl-1-azoniabicyclo[2.2.2]octan-2-yl](quinolin-4-yl)methanol bromide (I) Fractional atomic coordinates and isotropic or equivalent isotropic displacement parameters (Å^2^)

**Table:**

	*x*	*y*	*z*	*U*_iso_*/*U*_eq_	
Br1	0.75057 (3)	0.27380 (4)	0.57880 (3)	0.03605 (9)	
O1	0.5115 (2)	0.2121 (3)	0.6521 (3)	0.0362 (6)	
H1	0.572 (5)	0.209 (5)	0.642 (4)	0.054*	
N1	0.2489 (2)	0.3098 (2)	0.5310 (2)	0.0221 (5)	
C15	0.4907 (4)	0.6939 (4)	0.7102 (3)	0.0306 (8)	
H15	0.445324	0.659535	0.633537	0.037*	
C14	0.5537 (3)	0.5893 (4)	0.7985 (3)	0.0284 (6)	
C19	0.6229 (4)	0.6432 (5)	0.9122 (3)	0.0403 (8)	
N2	0.6882 (3)	0.5531 (4)	1.0046 (3)	0.0514 (9)	
C12	0.6235 (3)	0.3448 (4)	0.8711 (3)	0.0412 (8)	
H12	0.628646	0.241104	0.860850	0.049*	
C6	0.3120 (3)	0.3024 (4)	0.7490 (3)	0.0296 (7)	
H6A	0.369452	0.219011	0.781731	0.035*	
H6B	0.323150	0.376529	0.811878	0.035*	
C2	0.0924 (3)	0.3675 (4)	0.6283 (3)	0.0352 (7)	
H2	0.005740	0.328351	0.603565	0.042*	
C3	0.1782 (3)	0.2461 (4)	0.7058 (3)	0.0336 (9)	
H3	0.151610	0.221515	0.773899	0.040*	
C1	0.1254 (3)	0.3847 (4)	0.5157 (3)	0.0270 (6)	
H1A	0.059424	0.339538	0.448607	0.032*	
H1B	0.130621	0.491206	0.498492	0.032*	
C21	0.1927 (3)	0.3134 (3)	0.3070 (3)	0.0299 (7)	
C13	0.5546 (3)	0.4325 (3)	0.7787 (3)	0.0280 (6)	
C26	0.1140 (3)	0.4260 (4)	0.2454 (3)	0.0372 (7)	
H26	0.120294	0.522060	0.278633	0.045*	
C22	0.1858 (3)	0.1739 (4)	0.2559 (3)	0.0347 (7)	
H22	0.240605	0.097563	0.296478	0.042*	
C5	0.2326 (3)	0.1423 (4)	0.5392 (3)	0.0306 (6)	
H5A	0.314362	0.092895	0.561339	0.037*	
H5B	0.179902	0.103515	0.461868	0.037*	
C25	0.0260 (4)	0.3983 (5)	0.1352 (3)	0.0470 (9)	
H25	−0.029164	0.474416	0.094748	0.056*	
C4	0.1721 (3)	0.1082 (4)	0.6311 (3)	0.0386 (8)	
H4A	0.084569	0.078650	0.591521	0.046*	
H4B	0.216071	0.025160	0.681528	0.046*	
C24	0.0193 (3)	0.2582 (7)	0.0847 (3)	0.0493 (10)	
H24	−0.039309	0.239546	0.009033	0.059*	
C20	0.2904 (3)	0.3471 (3)	0.4266 (2)	0.0263 (6)	
H20A	0.311685	0.453674	0.429950	0.032*	
H20B	0.366413	0.290121	0.434368	0.032*	
C23	0.0985 (4)	0.1463 (5)	0.1450 (3)	0.0449 (9)	
H23	0.093245	0.050806	0.110940	0.054*	
C9	0.0952 (3)	0.5078 (5)	0.6977 (3)	0.0456 (9)	
H9	0.068495	0.496829	0.762940	0.055*	
C10	0.1296 (4)	0.6435 (5)	0.6806 (3)	0.0477 (10)	
H10A	0.157439	0.662902	0.616954	0.057*	
H10B	0.126306	0.721337	0.731969	0.057*	
C11	0.6868 (4)	0.4112 (5)	0.9821 (3)	0.0505 (10)	
H11	0.731413	0.347556	1.044541	0.061*	
C7	0.3415 (3)	0.3732 (3)	0.6463 (2)	0.0225 (5)	
H7	0.321988	0.481092	0.647173	0.027*	
C18	0.6270 (5)	0.7994 (6)	0.9329 (4)	0.0593 (13)	
H18	0.674977	0.836793	1.007814	0.071*	
C16	0.4942 (5)	0.8438 (5)	0.7334 (4)	0.0467 (11)	
H16	0.450616	0.911654	0.673689	0.056*	
C8	0.4806 (3)	0.3632 (4)	0.6601 (3)	0.0262 (7)	
H8	0.495513	0.420840	0.595927	0.031*	
C17	0.5632 (6)	0.8951 (5)	0.8469 (5)	0.0666 (14)	
H17	0.565128	0.998027	0.863411	0.080*	

### (R)-[(2S,4S,5R)-1-Benzyl-5-ethenyl-1-azoniabicyclo[2.2.2]octan-2-yl](quinolin-4-yl)methanol bromide (I) Atomic displacement parameters (Å^2^)

**Table:**

	*U*^11^	*U*^22^	*U*^33^	*U*^12^	*U*^13^	*U*^23^
Br1	0.03099 (14)	0.02947 (13)	0.05035 (18)	−0.00130 (17)	0.01742 (12)	−0.00135 (18)
O1	0.0240 (12)	0.0280 (11)	0.0565 (16)	0.0028 (10)	0.0135 (12)	−0.0032 (10)
N1	0.0179 (10)	0.0253 (15)	0.0227 (11)	−0.0007 (8)	0.0066 (8)	0.0006 (8)
C15	0.0284 (18)	0.031 (2)	0.0341 (17)	−0.0009 (14)	0.0123 (14)	0.0020 (14)
C14	0.0234 (15)	0.0352 (17)	0.0298 (15)	−0.0040 (12)	0.0132 (12)	−0.0003 (12)
C19	0.044 (2)	0.048 (2)	0.0326 (18)	−0.0106 (17)	0.0173 (16)	−0.0027 (16)
N2	0.053 (2)	0.064 (2)	0.0288 (15)	−0.0178 (17)	0.0041 (14)	0.0011 (15)
C12	0.0271 (16)	0.0372 (17)	0.049 (2)	−0.0009 (13)	−0.0001 (15)	0.0110 (15)
C6	0.0229 (12)	0.039 (2)	0.0255 (13)	−0.0011 (12)	0.0070 (10)	0.0034 (12)
C2	0.0208 (14)	0.054 (2)	0.0326 (16)	−0.0013 (14)	0.0121 (13)	0.0024 (15)
C3	0.0282 (14)	0.048 (3)	0.0263 (14)	−0.0076 (14)	0.0113 (11)	0.0053 (13)
C1	0.0177 (13)	0.0355 (16)	0.0262 (14)	0.0008 (11)	0.0055 (11)	0.0019 (12)
C21	0.0299 (14)	0.038 (2)	0.0239 (14)	−0.0055 (12)	0.0118 (12)	−0.0017 (11)
C13	0.0181 (13)	0.0314 (15)	0.0339 (16)	−0.0016 (11)	0.0080 (12)	0.0022 (12)
C26	0.0406 (19)	0.0417 (19)	0.0284 (16)	−0.0056 (15)	0.0108 (14)	0.0032 (14)
C22	0.0297 (17)	0.045 (2)	0.0332 (17)	−0.0022 (14)	0.0159 (14)	−0.0032 (14)
C5	0.0283 (16)	0.0277 (16)	0.0336 (16)	−0.0049 (12)	0.0076 (13)	−0.0008 (12)
C25	0.042 (2)	0.064 (3)	0.0294 (18)	−0.0018 (19)	0.0049 (16)	0.0106 (17)
C4	0.0350 (18)	0.0391 (17)	0.0407 (18)	−0.0134 (14)	0.0118 (15)	0.0053 (14)
C24	0.0456 (18)	0.075 (3)	0.0235 (14)	−0.015 (2)	0.0069 (13)	−0.004 (2)
C20	0.0255 (14)	0.0305 (15)	0.0243 (14)	−0.0024 (11)	0.0105 (11)	0.0001 (11)
C23	0.049 (2)	0.055 (2)	0.0354 (19)	−0.0148 (19)	0.0204 (18)	−0.0161 (17)
C9	0.0385 (19)	0.067 (3)	0.0357 (19)	0.0168 (19)	0.0185 (15)	0.0017 (17)
C10	0.044 (2)	0.057 (2)	0.040 (2)	0.0177 (19)	0.0119 (17)	−0.0095 (17)
C11	0.037 (2)	0.062 (3)	0.038 (2)	−0.0084 (18)	−0.0051 (16)	0.0168 (18)
C7	0.0172 (12)	0.0271 (14)	0.0218 (13)	−0.0016 (10)	0.0047 (10)	−0.0019 (11)
C18	0.086 (3)	0.056 (3)	0.0393 (19)	−0.021 (2)	0.026 (2)	−0.023 (2)
C16	0.055 (3)	0.034 (2)	0.056 (3)	0.0037 (19)	0.026 (2)	0.0030 (19)
C8	0.0188 (15)	0.0255 (16)	0.0340 (16)	−0.0011 (11)	0.0088 (12)	0.0008 (12)
C17	0.104 (4)	0.036 (2)	0.067 (3)	−0.012 (3)	0.040 (3)	−0.019 (2)

### (R)-[(2S,4S,5R)-1-Benzyl-5-ethenyl-1-azoniabicyclo[2.2.2]octan-2-yl](quinolin-4-yl)methanol bromide (I) Geometric parameters (Å, º)

**Table:**

O1—H1	0.73 (5)	C21—C20	1.500 (4)
O1—C8	1.392 (4)	C13—C8	1.501 (4)
N1—C1	1.494 (4)	C26—H26	0.9300
N1—C5	1.500 (4)	C26—C25	1.366 (5)
N1—C20	1.506 (4)	C22—H22	0.9300
N1—C7	1.517 (3)	C22—C23	1.369 (5)
C15—H15	0.9300	C5—H5A	0.9700
C15—C14	1.397 (5)	C5—H5B	0.9700
C15—C16	1.353 (5)	C5—C4	1.505 (5)
C14—C19	1.393 (5)	C25—H25	0.9300
C14—C13	1.407 (4)	C25—C24	1.368 (7)
C19—N2	1.352 (5)	C4—H4A	0.9700
C19—C18	1.401 (6)	C4—H4B	0.9700
N2—C11	1.282 (6)	C24—H24	0.9300
C12—H12	0.9300	C24—C23	1.360 (6)
C12—C13	1.351 (4)	C20—H20A	0.9700
C12—C11	1.397 (5)	C20—H20B	0.9700
C6—H6A	0.9700	C23—H23	0.9300
C6—H6B	0.9700	C9—H9	0.9300
C6—C3	1.499 (4)	C9—C10	1.299 (6)
C6—C7	1.510 (4)	C10—H10A	0.9300
C2—H2	0.9800	C10—H10B	0.9300
C2—C3	1.524 (5)	C11—H11	0.9300
C2—C1	1.517 (4)	C7—H7	0.9800
C2—C9	1.485 (5)	C7—C8	1.519 (4)
C3—H3	0.9800	C18—H18	0.9300
C3—C4	1.497 (5)	C18—C17	1.332 (7)
C1—H1A	0.9700	C16—H16	0.9300
C1—H1B	0.9700	C16—C17	1.383 (7)
C21—C26	1.366 (5)	C8—H8	0.9800
C21—C22	1.366 (5)	C17—H17	0.9300
			
C8—O1—H1	108 (4)	N1—C5—H5B	109.7
C1—N1—C5	108.4 (2)	N1—C5—C4	110.0 (3)
C1—N1—C20	110.0 (2)	H5A—C5—H5B	108.2
C1—N1—C7	105.5 (2)	C4—C5—H5A	109.7
C5—N1—C20	110.4 (2)	C4—C5—H5B	109.7
C5—N1—C7	111.6 (2)	C26—C25—H25	120.1
C20—N1—C7	110.7 (2)	C26—C25—C24	119.7 (4)
C14—C15—H15	119.2	C24—C25—H25	120.1
C16—C15—H15	119.2	C3—C4—C5	109.2 (3)
C16—C15—C14	121.6 (4)	C3—C4—H4A	109.8
C15—C14—C13	123.9 (3)	C3—C4—H4B	109.8
C19—C14—C15	118.3 (3)	C5—C4—H4A	109.8
C19—C14—C13	117.8 (3)	C5—C4—H4B	109.8
C14—C19—C18	118.8 (4)	H4A—C4—H4B	108.3
N2—C19—C14	123.6 (4)	C25—C24—H24	120.0
N2—C19—C18	117.5 (3)	C23—C24—C25	120.0 (3)
C11—N2—C19	116.3 (3)	C23—C24—H24	120.0
C13—C12—H12	120.3	N1—C20—H20A	108.8
C13—C12—C11	119.5 (4)	N1—C20—H20B	108.8
C11—C12—H12	120.3	C21—C20—N1	113.9 (2)
H6A—C6—H6B	108.2	C21—C20—H20A	108.8
C3—C6—H6A	109.7	C21—C20—H20B	108.8
C3—C6—H6B	109.7	H20A—C20—H20B	107.7
C3—C6—C7	109.6 (2)	C22—C23—H23	119.9
C7—C6—H6A	109.7	C24—C23—C22	120.1 (4)
C7—C6—H6B	109.7	C24—C23—H23	119.9
C3—C2—H2	106.9	C2—C9—H9	115.2
C1—C2—H2	106.9	C10—C9—C2	129.5 (4)
C1—C2—C3	108.0 (3)	C10—C9—H9	115.2
C9—C2—H2	106.9	C9—C10—H10A	120.0
C9—C2—C3	111.4 (3)	C9—C10—H10B	120.0
C9—C2—C1	116.3 (3)	H10A—C10—H10B	120.0
C6—C3—C2	109.0 (3)	N2—C11—C12	125.1 (3)
C6—C3—H3	110.3	N2—C11—H11	117.5
C2—C3—H3	110.3	C12—C11—H11	117.5
C4—C3—C6	108.1 (3)	N1—C7—H7	106.4
C4—C3—C2	108.9 (3)	N1—C7—C8	116.0 (2)
C4—C3—H3	110.3	C6—C7—N1	107.8 (2)
N1—C1—C2	110.4 (2)	C6—C7—H7	106.4
N1—C1—H1A	109.6	C6—C7—C8	113.2 (2)
N1—C1—H1B	109.6	C8—C7—H7	106.4
C2—C1—H1A	109.6	C19—C18—H18	119.5
C2—C1—H1B	109.6	C17—C18—C19	121.0 (4)
H1A—C1—H1B	108.1	C17—C18—H18	119.5
C26—C21—C20	119.6 (3)	C15—C16—H16	120.4
C22—C21—C26	119.5 (3)	C15—C16—C17	119.2 (5)
C22—C21—C20	120.8 (3)	C17—C16—H16	120.4
C14—C13—C8	121.8 (3)	O1—C8—C13	112.7 (3)
C12—C13—C14	117.6 (3)	O1—C8—C7	108.8 (3)
C12—C13—C8	120.5 (3)	O1—C8—H8	109.4
C21—C26—H26	119.8	C13—C8—C7	107.2 (3)
C25—C26—C21	120.5 (4)	C13—C8—H8	109.4
C25—C26—H26	119.8	C7—C8—H8	109.4
C21—C22—H22	119.9	C18—C17—C16	121.0 (4)
C21—C22—C23	120.1 (4)	C18—C17—H17	119.5
C23—C22—H22	119.9	C16—C17—H17	119.5
N1—C5—H5A	109.7		
			
N1—C5—C4—C3	−14.4 (4)	C21—C26—C25—C24	−2.0 (6)
N1—C7—C8—O1	−58.3 (4)	C21—C22—C23—C24	0.9 (5)
N1—C7—C8—C13	179.6 (2)	C13—C14—C19—N2	−1.5 (5)
C15—C14—C19—N2	−179.6 (3)	C13—C14—C19—C18	177.8 (4)
C15—C14—C19—C18	−0.4 (5)	C13—C12—C11—N2	−1.8 (6)
C15—C14—C13—C12	177.5 (3)	C26—C21—C22—C23	−1.6 (5)
C15—C14—C13—C8	−3.4 (5)	C26—C21—C20—N1	95.4 (3)
C15—C16—C17—C18	0.6 (8)	C26—C25—C24—C23	1.3 (6)
C14—C15—C16—C17	1.0 (8)	C22—C21—C26—C25	2.2 (5)
C14—C19—N2—C11	1.8 (6)	C22—C21—C20—N1	−88.2 (4)
C14—C19—C18—C17	1.9 (7)	C5—N1—C1—C2	67.6 (3)
C14—C13—C8—O1	169.2 (3)	C5—N1—C20—C21	66.4 (3)
C14—C13—C8—C7	−71.2 (4)	C5—N1—C7—C6	−43.7 (3)
C19—C14—C13—C12	−0.5 (5)	C5—N1—C7—C8	84.4 (3)
C19—C14—C13—C8	178.6 (3)	C25—C24—C23—C22	−0.7 (6)
C19—N2—C11—C12	−0.1 (6)	C20—N1—C1—C2	−171.6 (2)
C19—C18—C17—C16	−2.1 (8)	C20—N1—C5—C4	−171.2 (2)
N2—C19—C18—C17	−178.8 (5)	C20—N1—C7—C6	−167.1 (2)
C12—C13—C8—O1	−11.7 (4)	C20—N1—C7—C8	−39.0 (3)
C12—C13—C8—C7	107.9 (3)	C20—C21—C26—C25	178.7 (3)
C6—C3—C4—C5	−50.4 (4)	C20—C21—C22—C23	−178.1 (3)
C6—C7—C8—O1	67.1 (3)	C9—C2—C3—C6	−61.9 (3)
C6—C7—C8—C13	−55.1 (3)	C9—C2—C3—C4	−179.6 (3)
C2—C3—C4—C5	67.9 (3)	C9—C2—C1—N1	111.5 (3)
C3—C6—C7—N1	−21.9 (3)	C11—C12—C13—C14	2.0 (5)
C3—C6—C7—C8	−151.6 (3)	C11—C12—C13—C8	−177.1 (3)
C3—C2—C1—N1	−14.5 (4)	C7—N1—C1—C2	−52.1 (3)
C3—C2—C9—C10	119.5 (4)	C7—N1—C5—C4	65.2 (3)
C1—N1—C5—C4	−50.6 (3)	C7—N1—C20—C21	−169.5 (2)
C1—N1—C20—C21	−53.2 (3)	C7—C6—C3—C2	−45.7 (3)
C1—N1—C7—C6	73.9 (3)	C7—C6—C3—C4	72.5 (3)
C1—N1—C7—C8	−158.0 (2)	C18—C19—N2—C11	−177.5 (4)
C1—C2—C3—C6	66.9 (3)	C16—C15—C14—C19	−1.1 (6)
C1—C2—C3—C4	−50.8 (3)	C16—C15—C14—C13	−179.1 (4)
C1—C2—C9—C10	−4.8 (6)		

### (R)-[(2S,4S,5R)-1-Benzyl-5-ethenyl-1-azoniabicyclo[2.2.2]octan-2-yl](quinolin-4-yl)methanol bromide (I) Hydrogen-bond geometry (Å, º)

**Table:**

*D*—H···*A*	*D*—H	H···*A*	*D*···*A*	*D*—H···*A*
O1—H1···Br1	0.73 (5)	2.45 (5)	3.149 (3)	162 (5)
C15—H15···Br1^i^	0.93	2.90	3.644 (4)	137
C12—H12···O1	0.93	2.39	2.739 (5)	102
C6—H6*A*···O1	0.97	2.58	2.967 (4)	104
C2—H2···Br1^ii^	0.98	2.83	3.779 (3)	164
C26—H26···Br1^i^	0.93	2.87	3.738 (4)	155
C5—H5*A*···O1	0.97	2.36	3.024 (4)	125
C20—H20*A*···Br1^i^	0.97	2.91	3.800 (3)	153
C20—H20*B*···O1	0.97	2.64	3.198 (4)	117
C10—H10*A*···Br1^i^	0.93	3.02	3.943 (4)	172

Symmetry codes: (i) −*x*+1, *y*+1/2, −*z*+1; (ii) *x*−1, *y*, *z*.

### (R)-[(2S,4S,5R)-1-Benzyl-5-ethenyl-1-azoniabicyclo[2.2.2]octan-2-yl](quinolin-4-yl)methanol bromide sesquihydrate (II) Crystal data

**Table:**

2C_26_H_29_N_2_O^+^·2Br^−^·3H_2_O	*D*_x_ = 1.409 Mg m^−^^3^
*M**_r_* = 984.89	Mo *K*α radiation, λ = 0.71073 Å
Tetragonal, *P*4_1_2_1_2	Cell parameters from 19124 reflections
*a* = 9.9254 (2) Å	θ = 1.7–28.6°
*c* = 47.1267 (14) Å	µ = 1.80 mm^−^^1^
*V* = 4642.6 (2) Å^3^	*T* = 173 K
*Z* = 4	Block, colorless
*F*(000) = 2056	0.52 × 0.36 × 0.36 mm

### (R)-[(2S,4S,5R)-1-Benzyl-5-ethenyl-1-azoniabicyclo[2.2.2]octan-2-yl](quinolin-4-yl)methanol bromide sesquihydrate (II) Data collection

**Table:**

XtaLABmini diffractometer	3919 reflections with *I* > 2σ(*I*)
ω scans	*R*_int_ = 0.078
Absorption correction: multi-scan (CrysAlisPro; Rigaku OD, 2020)	θ_max_ = 25.1°, θ_min_ = 1.7°
*T*_min_ = 0.281, *T*_max_ = 1.000	*h* = −11→11
36339 measured reflections	*k* = −11→11
4154 independent reflections	*l* = −56→56

### (R)-[(2S,4S,5R)-1-Benzyl-5-ethenyl-1-azoniabicyclo[2.2.2]octan-2-yl](quinolin-4-yl)methanol bromide sesquihydrate (II) Refinement

**Table:**

Refinement on *F*^2^	Hydrogen site location: mixed
Least-squares matrix: full	H atoms treated by a mixture of independent and constrained refinement
*R*[*F*^2^ > 2σ(*F*^2^)] = 0.051	*w* = 1/[σ^2^(*F*_o_^2^) + 19.0784*P*] where *P* = (*F*_o_^2^ + 2*F*_c_^2^)/3
*wR*(*F*^2^) = 0.111	(Δ/σ)_max_ < 0.001
*S* = 1.05	Δρ_max_ = 0.35 e Å^−^^3^
4154 reflections	Δρ_min_ = −0.40 e Å^−^^3^
297 parameters	Absolute structure: Flack *x* determined using 1347 quotients [(*I*^+^)-(*I*^-^)]/[(*I*^+^)+(*I*^-^)] (Parsons et al., 2013)
0 restraints	Absolute structure parameter: 0.005 (7)

### (R)-[(2S,4S,5R)-1-Benzyl-5-ethenyl-1-azoniabicyclo[2.2.2]octan-2-yl](quinolin-4-yl)methanol bromide sesquihydrate (II) Special details

**Table:**

Geometry. All esds (except the esd in the dihedral angle between two l.s. planes) are estimated using the full covariance matrix. The cell esds are taken into account individually in the estimation of esds in distances, angles and torsion angles; correlations between esds in cell parameters are only used when they are defined by crystal symmetry. An approximate (isotropic) treatment of cell esds is used for estimating esds involving l.s. planes.

### (R)-[(2S,4S,5R)-1-Benzyl-5-ethenyl-1-azoniabicyclo[2.2.2]octan-2-yl](quinolin-4-yl)methanol bromide sesquihydrate (II) Fractional atomic coordinates and isotropic or equivalent isotropic displacement parameters (Å^2^)

**Table:**

	*x*	*y*	*z*	*U*_iso_*/*U*_eq_	
Br1	0.35481 (9)	0.04938 (9)	0.45637 (2)	0.0479 (2)	
O1	0.7691 (5)	0.4108 (5)	0.40753 (10)	0.0320 (12)	
H1	0.771 (8)	0.440 (8)	0.3897 (17)	0.048*	
O2	0.2790 (7)	−0.0285 (6)	0.39240 (11)	0.0490 (16)	
H2A	0.291 (10)	−0.117 (10)	0.3911 (19)	0.074*	
H2B	0.314 (10)	−0.004 (10)	0.406 (2)	0.074*	
N1	0.7332 (6)	0.2070 (5)	0.45114 (11)	0.0252 (12)	
N2	0.3561 (6)	0.6995 (5)	0.39911 (11)	0.0303 (13)	
C14	0.3953 (6)	0.4566 (7)	0.40458 (12)	0.0235 (14)	
C19	0.3097 (7)	0.5689 (7)	0.39967 (13)	0.0264 (15)	
C15	0.3366 (7)	0.3281 (6)	0.40504 (12)	0.0255 (14)	
H15	0.392546	0.251558	0.407842	0.031*	
C21	0.7978 (7)	−0.0296 (7)	0.43689 (13)	0.0272 (15)	
C8	0.6369 (6)	0.3692 (7)	0.41379 (12)	0.0243 (14)	
H8	0.612513	0.292345	0.401081	0.029*	
C18	0.1705 (7)	0.5476 (8)	0.39555 (13)	0.0344 (16)	
H18	0.113163	0.622347	0.392030	0.041*	
C13	0.5362 (7)	0.4828 (6)	0.40935 (12)	0.0238 (14)	
C7	0.6324 (7)	0.3211 (6)	0.44467 (11)	0.0222 (13)	
H7	0.540122	0.283909	0.448028	0.027*	
C26	0.7495 (7)	−0.1293 (7)	0.45498 (14)	0.0327 (15)	
H26	0.660524	−0.123764	0.462339	0.039*	
C12	0.5777 (7)	0.6140 (7)	0.40851 (13)	0.0276 (15)	
H12	0.670326	0.634744	0.411066	0.033*	
C3	0.7655 (7)	0.4006 (7)	0.48738 (13)	0.0293 (16)	
H3	0.778720	0.475896	0.501210	0.035*	
C2	0.7276 (7)	0.2713 (7)	0.50314 (13)	0.0293 (16)	
H2	0.808128	0.242861	0.514409	0.035*	
C1	0.6996 (7)	0.1598 (7)	0.48123 (12)	0.0271 (15)	
H1A	0.754331	0.079421	0.485869	0.033*	
H1B	0.603387	0.133791	0.482176	0.033*	
C16	0.2012 (7)	0.3101 (7)	0.40157 (14)	0.0307 (16)	
H16	0.163499	0.222222	0.402525	0.037*	
C11	0.4857 (8)	0.7178 (7)	0.40396 (14)	0.0325 (17)	
H11	0.518450	0.807669	0.404381	0.039*	
C5	0.8787 (7)	0.2519 (7)	0.45190 (14)	0.0301 (15)	
H5A	0.909847	0.271857	0.432393	0.036*	
H5B	0.935407	0.178419	0.459612	0.036*	
C17	0.1177 (7)	0.4223 (8)	0.39656 (14)	0.0347 (17)	
H17	0.023662	0.409823	0.393869	0.042*	
C6	0.6538 (8)	0.4363 (7)	0.46649 (13)	0.0304 (15)	
H6A	0.677762	0.520318	0.456348	0.036*	
H6B	0.568948	0.452397	0.476995	0.036*	
C4	0.8939 (7)	0.3778 (7)	0.47045 (14)	0.0340 (17)	
H4A	0.970945	0.366248	0.483558	0.041*	
H4B	0.912026	0.457167	0.458308	0.041*	
C9	0.6149 (9)	0.2958 (7)	0.52372 (14)	0.0383 (18)	
H9	0.639217	0.342463	0.540550	0.046*	
C20	0.7142 (7)	0.0920 (6)	0.43043 (12)	0.0279 (15)	
H20A	0.618000	0.065575	0.430394	0.034*	
H20B	0.736832	0.124169	0.411124	0.034*	
C24	0.9589 (9)	−0.2483 (8)	0.45077 (19)	0.052 (2)	
H24	1.013632	−0.323543	0.455513	0.062*	
C22	0.9260 (8)	−0.0440 (8)	0.42540 (16)	0.0422 (19)	
H22	0.958430	0.021589	0.412406	0.051*	
C25	0.8320 (9)	−0.2368 (7)	0.46221 (14)	0.043 (2)	
H25	0.800483	−0.303018	0.475171	0.052*	
C10	0.4874 (9)	0.2619 (8)	0.52178 (15)	0.042 (2)	
H10A	0.455852	0.214897	0.505520	0.051*	
H10B	0.426718	0.284260	0.536633	0.051*	
C23	1.0070 (9)	−0.1520 (9)	0.43257 (19)	0.051 (2)	
H23	1.095307	−0.159406	0.424949	0.062*	
O3	1.0704 (8)	0.0704 (8)	0.500000	0.068 (3)	
H3A	1.074 (12)	0.139 (11)	0.512 (2)	0.102*	

### (R)-[(2S,4S,5R)-1-Benzyl-5-ethenyl-1-azoniabicyclo[2.2.2]octan-2-yl](quinolin-4-yl)methanol bromide sesquihydrate (II) Atomic displacement parameters (Å^2^)

**Table:**

	*U*^11^	*U*^22^	*U*^33^	*U*^12^	*U*^13^	*U*^23^
Br1	0.0629 (6)	0.0556 (5)	0.0251 (3)	−0.0257 (4)	−0.0098 (4)	0.0106 (4)
O1	0.024 (2)	0.046 (3)	0.026 (2)	0.002 (2)	−0.001 (2)	0.012 (2)
O2	0.089 (5)	0.029 (3)	0.029 (3)	0.007 (3)	−0.015 (3)	0.005 (2)
N1	0.032 (3)	0.023 (3)	0.021 (3)	−0.001 (2)	−0.003 (2)	0.000 (2)
N2	0.042 (4)	0.026 (3)	0.022 (3)	0.006 (3)	−0.004 (3)	0.005 (2)
C14	0.031 (4)	0.030 (4)	0.010 (3)	0.004 (3)	−0.002 (2)	0.000 (3)
C19	0.034 (4)	0.028 (4)	0.017 (3)	0.006 (3)	−0.003 (3)	0.001 (3)
C15	0.033 (4)	0.027 (4)	0.016 (3)	0.002 (3)	−0.001 (3)	0.000 (3)
C21	0.037 (4)	0.029 (4)	0.015 (3)	0.005 (3)	−0.001 (3)	0.000 (3)
C8	0.026 (3)	0.031 (4)	0.015 (3)	0.001 (3)	0.000 (3)	0.005 (3)
C18	0.032 (4)	0.041 (4)	0.030 (3)	0.015 (4)	−0.002 (3)	0.001 (3)
C13	0.031 (4)	0.026 (4)	0.014 (3)	−0.001 (3)	−0.001 (3)	0.001 (2)
C7	0.029 (4)	0.028 (4)	0.010 (3)	0.002 (3)	0.002 (3)	0.001 (2)
C26	0.041 (4)	0.028 (4)	0.030 (3)	−0.004 (3)	0.001 (3)	−0.001 (3)
C12	0.033 (4)	0.029 (4)	0.020 (3)	−0.002 (3)	0.001 (3)	0.004 (3)
C3	0.041 (4)	0.026 (4)	0.021 (3)	−0.003 (3)	−0.009 (3)	0.000 (3)
C2	0.043 (4)	0.026 (4)	0.019 (3)	−0.002 (3)	−0.010 (3)	0.000 (3)
C1	0.041 (4)	0.027 (4)	0.014 (3)	0.001 (3)	−0.003 (3)	0.002 (3)
C16	0.034 (4)	0.035 (4)	0.023 (3)	−0.004 (3)	0.001 (3)	0.002 (3)
C11	0.049 (5)	0.025 (4)	0.024 (3)	−0.003 (3)	0.003 (3)	0.007 (3)
C5	0.027 (4)	0.034 (4)	0.028 (3)	0.001 (3)	−0.004 (3)	0.006 (3)
C17	0.027 (4)	0.046 (5)	0.031 (4)	0.004 (3)	−0.006 (3)	0.000 (3)
C6	0.045 (4)	0.026 (3)	0.021 (3)	0.002 (3)	−0.004 (3)	−0.001 (3)
C4	0.039 (4)	0.032 (4)	0.031 (4)	−0.007 (3)	−0.006 (3)	0.009 (3)
C9	0.060 (6)	0.033 (4)	0.022 (3)	−0.003 (4)	0.001 (3)	−0.008 (3)
C20	0.042 (4)	0.026 (4)	0.016 (3)	0.005 (3)	−0.004 (3)	0.000 (3)
C24	0.048 (5)	0.028 (4)	0.078 (6)	0.010 (4)	−0.021 (5)	−0.014 (4)
C22	0.052 (5)	0.032 (4)	0.042 (4)	0.000 (4)	0.013 (4)	0.000 (4)
C25	0.076 (6)	0.024 (4)	0.029 (4)	−0.003 (4)	−0.015 (4)	0.002 (3)
C10	0.063 (6)	0.041 (4)	0.023 (4)	−0.003 (4)	0.011 (3)	−0.004 (3)
C23	0.042 (5)	0.040 (5)	0.072 (6)	0.005 (4)	0.006 (4)	−0.013 (5)
O3	0.068 (4)	0.068 (4)	0.068 (7)	0.018 (6)	−0.006 (4)	0.006 (4)

### (R)-[(2S,4S,5R)-1-Benzyl-5-ethenyl-1-azoniabicyclo[2.2.2]octan-2-yl](quinolin-4-yl)methanol bromide sesquihydrate (II) Geometric parameters (Å, º)

**Table:**

O1—H1	0.89 (8)	C3—C6	1.524 (9)
O1—C8	1.406 (8)	C3—C4	1.521 (10)
O2—H2A	0.88 (10)	C2—H2	1.0000
O2—H2B	0.75 (9)	C2—C1	1.539 (9)
N1—C7	1.541 (8)	C2—C9	1.501 (10)
N1—C1	1.530 (8)	C1—H1A	0.9900
N1—C5	1.512 (8)	C1—H1B	0.9900
N1—C20	1.514 (8)	C16—H16	0.9500
N2—C19	1.376 (9)	C16—C17	1.409 (10)
N2—C11	1.319 (9)	C11—H11	0.9500
C14—C19	1.421 (9)	C5—H5A	0.9900
C14—C15	1.402 (9)	C5—H5B	0.9900
C14—C13	1.440 (9)	C5—C4	1.533 (9)
C19—C18	1.411 (10)	C17—H17	0.9500
C15—H15	0.9500	C6—H6A	0.9900
C15—C16	1.366 (10)	C6—H6B	0.9900
C21—C26	1.391 (9)	C4—H4A	0.9900
C21—C20	1.496 (9)	C4—H4B	0.9900
C21—C22	1.390 (10)	C9—H9	0.9500
C8—H8	1.0000	C9—C10	1.312 (11)
C8—C13	1.521 (9)	C20—H20A	0.9900
C8—C7	1.532 (7)	C20—H20B	0.9900
C18—H18	0.9500	C24—H24	0.9500
C18—C17	1.350 (11)	C24—C25	1.375 (12)
C13—C12	1.367 (9)	C24—C23	1.370 (12)
C7—H7	1.0000	C22—H22	0.9500
C7—C6	1.553 (8)	C22—C23	1.382 (12)
C26—H26	0.9500	C25—H25	0.9500
C26—C25	1.388 (10)	C10—H10A	0.9500
C12—H12	0.9500	C10—H10B	0.9500
C12—C11	1.393 (9)	C23—H23	0.9500
C3—H3	1.0000	O3—H3A	0.90 (10)
C3—C2	1.530 (9)	O3—H3A^i^	0.90 (10)
			
C8—O1—H1	109 (5)	N1—C1—H1A	109.4
H2A—O2—H2B	108 (9)	N1—C1—H1B	109.4
C1—N1—C7	105.5 (5)	C2—C1—H1A	109.4
C5—N1—C7	114.1 (5)	C2—C1—H1B	109.4
C5—N1—C1	106.0 (5)	H1A—C1—H1B	108.0
C5—N1—C20	110.9 (5)	C15—C16—H16	120.1
C20—N1—C7	110.2 (5)	C15—C16—C17	119.7 (7)
C20—N1—C1	109.8 (5)	C17—C16—H16	120.1
C11—N2—C19	116.9 (6)	N2—C11—C12	124.4 (7)
C19—C14—C13	117.7 (6)	N2—C11—H11	117.8
C15—C14—C19	117.9 (6)	C12—C11—H11	117.8
C15—C14—C13	124.4 (6)	N1—C5—H5A	109.6
N2—C19—C14	122.8 (6)	N1—C5—H5B	109.6
N2—C19—C18	117.8 (6)	N1—C5—C4	110.3 (5)
C18—C19—C14	119.4 (6)	H5A—C5—H5B	108.1
C14—C15—H15	119.1	C4—C5—H5A	109.6
C16—C15—C14	121.7 (6)	C4—C5—H5B	109.6
C16—C15—H15	119.1	C18—C17—C16	120.4 (7)
C26—C21—C20	120.5 (6)	C18—C17—H17	119.8
C22—C21—C26	118.8 (7)	C16—C17—H17	119.8
C22—C21—C20	120.7 (6)	C7—C6—H6A	109.5
O1—C8—H8	108.9	C7—C6—H6B	109.5
O1—C8—C13	111.5 (5)	C3—C6—C7	110.9 (5)
O1—C8—C7	108.5 (5)	C3—C6—H6A	109.5
C13—C8—H8	108.9	C3—C6—H6B	109.5
C13—C8—C7	110.0 (5)	H6A—C6—H6B	108.1
C7—C8—H8	108.9	C3—C4—C5	109.8 (6)
C19—C18—H18	119.5	C3—C4—H4A	109.7
C17—C18—C19	120.9 (7)	C3—C4—H4B	109.7
C17—C18—H18	119.5	C5—C4—H4A	109.7
C14—C13—C8	121.7 (6)	C5—C4—H4B	109.7
C12—C13—C14	117.4 (6)	H4A—C4—H4B	108.2
C12—C13—C8	120.8 (6)	C2—C9—H9	115.4
N1—C7—H7	107.0	C10—C9—C2	129.2 (7)
N1—C7—C6	108.7 (5)	C10—C9—H9	115.4
C8—C7—N1	113.5 (5)	N1—C20—H20A	108.7
C8—C7—H7	107.0	N1—C20—H20B	108.7
C8—C7—C6	113.3 (5)	C21—C20—N1	114.1 (5)
C6—C7—H7	107.0	C21—C20—H20A	108.7
C21—C26—H26	120.2	C21—C20—H20B	108.7
C25—C26—C21	119.6 (7)	H20A—C20—H20B	107.6
C25—C26—H26	120.2	C25—C24—H24	119.8
C13—C12—H12	119.6	C23—C24—H24	119.8
C13—C12—C11	120.8 (7)	C23—C24—C25	120.4 (7)
C11—C12—H12	119.6	C21—C22—H22	119.4
C2—C3—H3	110.1	C23—C22—C21	121.1 (8)
C6—C3—H3	110.1	C23—C22—H22	119.4
C6—C3—C2	109.3 (6)	C26—C25—H25	119.7
C4—C3—H3	110.1	C24—C25—C26	120.5 (7)
C4—C3—C2	109.6 (6)	C24—C25—H25	119.7
C4—C3—C6	107.8 (5)	C9—C10—H10A	120.0
C3—C2—H2	107.4	C9—C10—H10B	120.0
C3—C2—C1	108.8 (5)	H10A—C10—H10B	120.0
C1—C2—H2	107.4	C24—C23—C22	119.4 (8)
C9—C2—C3	111.1 (6)	C24—C23—H23	120.3
C9—C2—H2	107.4	C22—C23—H23	120.3
C9—C2—C1	114.5 (6)	H3A—O3—H3A^i^	110 (10)
N1—C1—C2	111.2 (5)		
			
O1—C8—C13—C14	157.9 (5)	C26—C21—C20—N1	88.3 (8)
O1—C8—C13—C12	−19.2 (8)	C26—C21—C22—C23	−2.6 (11)
O1—C8—C7—N1	−56.9 (7)	C3—C2—C1—N1	6.4 (8)
O1—C8—C7—C6	67.7 (7)	C3—C2—C9—C10	105.2 (9)
N1—C7—C6—C3	−0.5 (7)	C2—C3—C6—C7	−59.5 (7)
N1—C5—C4—C3	12.3 (7)	C2—C3—C4—C5	52.0 (7)
N2—C19—C18—C17	−178.3 (6)	C1—N1—C7—C8	−172.1 (5)
C14—C19—C18—C17	0.8 (10)	C1—N1—C7—C6	60.9 (6)
C14—C15—C16—C17	1.9 (10)	C1—N1—C5—C4	−66.9 (6)
C14—C13—C12—C11	1.7 (9)	C1—N1—C20—C21	−58.1 (7)
C19—N2—C11—C12	2.4 (10)	C1—C2—C9—C10	−18.5 (12)
C19—C14—C15—C16	−1.5 (9)	C11—N2—C19—C14	−1.3 (9)
C19—C14—C13—C8	−177.9 (5)	C11—N2—C19—C18	177.7 (6)
C19—C14—C13—C12	−0.7 (8)	C5—N1—C7—C8	71.9 (6)
C19—C18—C17—C16	−0.4 (10)	C5—N1—C7—C6	−55.1 (6)
C15—C14—C19—N2	179.2 (6)	C5—N1—C1—C2	56.0 (7)
C15—C14—C19—C18	0.2 (9)	C5—N1—C20—C21	58.7 (7)
C15—C14—C13—C8	3.5 (9)	C6—C3—C2—C1	55.6 (7)
C15—C14—C13—C12	−179.3 (6)	C6—C3—C2—C9	−71.4 (7)
C15—C16—C17—C18	−0.9 (10)	C6—C3—C4—C5	−66.8 (7)
C21—C26—C25—C24	−2.6 (11)	C4—C3—C2—C1	−62.2 (7)
C21—C22—C23—C24	1.6 (13)	C4—C3—C2—C9	170.8 (6)
C8—C13—C12—C11	178.9 (6)	C4—C3—C6—C7	59.5 (7)
C8—C7—C6—C3	−127.6 (6)	C9—C2—C1—N1	131.5 (6)
C13—C14—C19—N2	0.5 (9)	C20—N1—C7—C8	−53.6 (7)
C13—C14—C19—C18	−178.6 (6)	C20—N1—C7—C6	179.4 (5)
C13—C14—C15—C16	177.1 (6)	C20—N1—C1—C2	175.9 (5)
C13—C8—C7—N1	−179.2 (5)	C20—N1—C5—C4	173.9 (5)
C13—C8—C7—C6	−54.6 (7)	C20—C21—C26—C25	−175.7 (6)
C13—C12—C11—N2	−2.7 (10)	C20—C21—C22—C23	176.1 (7)
C7—N1—C1—C2	−65.4 (6)	C22—C21—C26—C25	3.1 (10)
C7—N1—C5—C4	48.7 (7)	C22—C21—C20—N1	−90.4 (8)
C7—N1—C20—C21	−173.9 (5)	C25—C24—C23—C22	−1.1 (13)
C7—C8—C13—C14	−81.6 (7)	C23—C24—C25—C26	1.6 (12)
C7—C8—C13—C12	101.3 (7)		

Symmetry code: (i) *y*+1, *x*−1, −*z*+1.

### (R)-[(2S,4S,5R)-1-Benzyl-5-ethenyl-1-azoniabicyclo[2.2.2]octan-2-yl](quinolin-4-yl)methanol bromide sesquihydrate (II) Hydrogen-bond geometry (Å, º)

**Table:**

*D*—H···*A*	*D*—H	H···*A*	*D*···*A*	*D*—H···*A*
O1—H1···O2^ii^	0.89 (8)	1.75 (8)	2.629 (7)	168 (8)
O2—H2*A*···N2^iii^	0.88 (10)	1.97 (10)	2.824 (8)	161 (9)
O2—H2*B*···Br1	0.75 (9)	2.48 (9)	3.202 (5)	160 (10)
C15—H15···Br1	0.95	3.07	3.679 (6)	124
C15—H15···O2	0.95	3.09	3.634 (9)	118
C7—H7···Br1	1.00	2.99	3.894 (6)	151
C12—H12···O1	0.95	2.44	2.771 (8)	101
C12—H12···O2^ii^	0.95	2.94	3.236 (9)	100
C3—H3···Br1^iv^	1.00	3.56	3.895 (6)	102
C2—H2···Br1^iv^	1.00	2.98	3.811 (7)	142
C2—H2···O3	1.00	3.19	3.946 (8)	134
C1—H1*A*···O3	0.99	3.21	3.888 (9)	127
C1—H1*B*···Br1	0.99	2.88	3.779 (7)	152
C16—H16···O1^v^	0.95	3.29	3.608 (9)	102
C16—H16···O2	0.95	2.78	3.475 (9)	131
C11—H11···O2^vi^	0.95	2.93	3.293 (9)	104
C5—H5*A*···O1	0.99	2.29	2.836 (8)	114
C5—H5*B*···O3	0.99	2.56	3.464 (6)	151
C17—H17···O1^vii^	0.95	2.61	3.500 (8)	157
C17—H17···O2^v^	0.95	3.19	3.973 (10)	140
C6—H6*A*···O1	0.99	2.70	3.016 (8)	99
C4—H4*A*···Br1^iv^	0.99	2.94	3.785 (7)	144
C4—H4*A*···O3	0.99	3.19	3.784 (8)	120
C4—H4*B*···O1	0.99	2.82	3.230 (8)	106
C9—H9···N2^viii^	0.95	2.91	3.780 (9)	153
C20—H20*A*···Br1	0.99	2.89	3.794 (7)	152
C20—H20*B*···O1	0.99	2.87	3.387 (8)	114
C22—H22···O2^ix^	0.95	3.36	3.837 (11)	114
C10—H10*A*···Br1	0.95	3.01	3.960 (8)	176
C23—H23···O2^ix^	0.95	2.71	3.518 (11)	143
O3—H3*A*···Br1^iv^	0.90 (10)	2.61 (10)	3.499 (6)	170 (11)

Symmetry codes: (ii) *x*+1/2, −*y*+1/2, −*z*+3/4; (iii) *x*, *y*−1, *z*; (iv) *y*+1, *x*, −*z*+1; (v) *x*−1/2, −*y*+1/2, −*z*+3/4; (vi) *x*, *y*+1, *z*; (vii) *x*−1, *y*, *z*; (viii) *y*, *x*, −*z*+1; (ix) *x*+1, *y*, *z*.
